# Supplementary material for: The Systems Biology Research Tool: evolvable open-source software
Source: BMC Syst Biol. 2008 Jun 29;2:55. doi: 10.1186/1752-0509-2-55 (PMC2446383; doi:10.1186/1752-0509-2-55)
Supplement: Additional file 1 — SBRT Archive. An archive of the current version of the Systems Biology Research Tool. [file 1752-0509-2-55-S1.zip › sbrt-1.4.0/doc/developers_guide/api/sbrt/kernel/fba/proc/FluxPlasticityProcess.html]

FluxPlasticityProcess


|  |  |  |
| --- | --- | --- |
| **Overview** \| **Package** \| **Class** \| **Use** \| **Tree** \| **Deprecated** \| **Index** \| **Help** | |  |
| **PREV CLASS**   **NEXT CLASS** | **FRAMES**    **NO FRAMES**     **All Classes** |  |
| SUMMARY: NESTED \| FIELD \| CONSTR \| METHOD | DETAIL: FIELD \| CONSTR \| METHOD |  |


---


## sbrt.kernel.fba.proc Class FluxPlasticityProcess

```
java.lang.Object
  sbrt.kernel.fba.proc.FbaProcess<FluxPlasticityProcess.Results>
      sbrt.kernel.fba.proc.FluxPlasticityProcess
```

**All Implemented Interfaces:**: KernelProcess<FluxPlasticityProcess.Results>, MonitoredProcess<FluxPlasticityProcess.Results>

---

``` public final class FluxPlasticityProcess extends FbaProcess<FluxPlasticityProcess.Results> implements MonitoredProcess<FluxPlasticityProcess.Results> ```

This class is used to perform flux plasticity experiments.

**Author:**
:   This class was written and documented by Jeremiah Wright
    while in the Wagner lab.

---

| **Nested Class Summary** | |
| --- | --- |
| `static class` | `FluxPlasticityProcess.Results`             This class is used to represent the results of a flux plasticity experiment. |


**Constructor Summary**

`FluxPlasticityProcess(Fluxome fluxome)`
  
          Constructs a new flux plasticity experiment.


| **Method Summary** | |
| --- | --- |
| `Fluxome` | `getFluxome()`             Returns the fluxome used in this flux plasticity experiment. |
| `ProgressMonitor` | `getProgress()`             Returns a progress monitor for this experiment. |
| `void` | `run(ResultsManager<FluxPlasticityProcess.Results> resultsManager)`             Iterates thru the flux range source set via `setFluxRangeSource(Iterable)`, computes the flux plasticity measures, and adds the results of each to the provided results manager. |
| `void` | `setFluxIntervalSource(java.lang.Iterable<java.util.Map<java.lang.String,Interval>> intervalSource)`             Sets the flux interval source. |
| `void` | `setZeroCutoff(double zeroCutoff)`             Sets the zero cutoff to the provided value. |

**Methods inherited from class sbrt.kernel.fba.proc.FbaProcess**

`toString`

**Methods inherited from class java.lang.Object**

`clone, equals, finalize, getClass, hashCode, notify, notifyAll, wait, wait, wait`

**Constructor Detail**

### FluxPlasticityProcess

```
public FluxPlasticityProcess(Fluxome fluxome)
```

:   Constructs a new flux plasticity experiment.

    **Parameters:**: `fluxome` - the fluxome for which flux plasticities will be determined.


**Method Detail**

### getFluxome

```
public Fluxome getFluxome()
```

:   Returns the fluxome used in this flux plasticity experiment.

    :   **Specified by:**: `getFluxome` in class `FbaProcess<FluxPlasticityProcess.Results>`
    :   **Returns:**: the fluxome used in this flux plasticity experiment.

---


### getProgress

```
public ProgressMonitor getProgress()
```

:   Returns a progress monitor for this experiment.

    :   **Specified by:**: `getProgress` in interface `MonitoredProcess<FluxPlasticityProcess.Results>`
    :   **Returns:**: a progress monitor for this experiment.

---


### setFluxIntervalSource

```
public void setFluxIntervalSource(java.lang.Iterable<java.util.Map<java.lang.String,Interval>> intervalSource)
```

:   Sets the flux interval source. These are the flux intervals for which
    flux plasticities will be measured.

    :   **Parameters:**: `intervalSource` - a source of flux ranges, or constraints.

---


### setZeroCutoff

```
public void setZeroCutoff(double zeroCutoff)
```

:   Sets the zero cutoff to the provided value. The zero cutoff is the value
    used to decide which computed values are close enough to zero to be
    considered equal to zero. This value is an upper bound and should itself
    be close to zero, like 1E-6 for example.

    :   **Parameters:**: `zeroCutoff` - the zero cutoff.

---


### run

```
public void run(ResultsManager<FluxPlasticityProcess.Results> resultsManager)
```

:   Iterates thru the flux range source set via
    `setFluxRangeSource(Iterable)`, computes the flux plasticity
    measures, and adds the results of each to the
    provided results manager.

    :   **Specified by:**: `run` in interface `KernelProcess<FluxPlasticityProcess.Results>`
    :   **Parameters:**: `resultsManager` - the results manager to which each set of results is added.


---


|  |  |  |
| --- | --- | --- |
| **Overview** \| **Package** \| **Class** \| **Use** \| **Tree** \| **Deprecated** \| **Index** \| **Help** | |  |
| **PREV CLASS**   **NEXT CLASS** | **FRAMES**    **NO FRAMES**     **All Classes** |  |
| SUMMARY: NESTED \| FIELD \| CONSTR \| METHOD | DETAIL: FIELD \| CONSTR \| METHOD |  |


---
